# Tubular Structure Induced by a Plant Virus Facilitates Viral Spread in Its Vector Insect

**DOI:** 10.1371/journal.ppat.1003032

**Published:** 2012-11-15

**Authors:** Qian Chen, Hongyan Chen, Qianzhuo Mao, Qifei Liu, Takumi Shimizu, Tamaki Uehara-Ichiki, Zujian Wu, Lianhui Xie, Toshihiro Omura, Taiyun Wei

**Affiliations:** 1 Fujian Province Key Laboratory of Plant Virology, Institute of Plant Virology, Fujian Agriculture and Forestry University, Fuzhou, Fujian, PR China; 2 National Agricultural Research Center, Tsukuba, Ibaraki, Japan; University of Kentucky, United States of America

## Abstract

*Rice dwarf virus* (RDV) replicates in and is transmitted by a leafhopper vector in a persistent-propagative manner. Previous cytopathologic and genetic data revealed that tubular structures, constructed by the nonstructural viral protein Pns10, contain viral particles and are directly involved in the intercellular spread of RDV among cultured leafhopper cells. Here, we demonstrated that RDV exploited these virus-containing tubules to move along actin-based microvilli of the epithelial cells and muscle fibers of visceral muscle tissues in the alimentary canal, facilitating the spread of virus in the body of its insect vector leafhoppers. In cultured leafhopper cells, the knockdown of Pns10 expression due to RNA interference (RNAi) induced by synthesized dsRNA from Pns10 gene strongly inhibited tubule formation and prevented the spread of virus among insect vector cells. RNAi induced after ingestion of dsRNA from Pns10 gene strongly inhibited formation of tubules, preventing intercellular spread and transmission of the virus by the leafhopper. All these results, for the first time, show that a persistent-propagative virus exploits virus-containing tubules composed of a nonstructural viral protein to traffic along actin-based cellular protrusions, facilitating the intercellular spread of the virus in the vector insect. The RNAi strategy and the insect vector cell culture provide useful tools to investigate the molecular mechanisms enabling efficient transmission of persistent-propagative plant viruses by vector insects.

## Author Summary

Numerous plant viruses that seriously damage agricultural crops are transmitted by insects. However, the mechanisms enabling virus transmission by vector insects have been poorly understood, in part, due to the lack of useful tools. A persistent-propagative plant virus replicates and encodes nonstructural proteins to form various cytopathological structures in their two types of hosts: plants and vector insects. Here, we took advantage of unique biological tools, including insect vector cell culture and RNA interference (RNAi) induced by synthesized dsRNA, to investigate the molecular mechanisms facilitating the efficient spread of *Rice dwarf virus* (RDV), a persistent-propagative plant virus, among cells and organs of leafhopper vector. Our experimental evidence shows that RDV exploited virus-containing tubules composed of nonstructural viral protein Pns10 to traffic along actin-based cellular machinery, allowing efficient cell-to-cell spread of the virus in leafhopper vector. Consistently, and in support of a function of Pns10 tubules as a determinant for viral spread in vector insect, the introduction of dsRNA from Pns10 gene into cultured insect vector cells or intact insect strongly inhibited such tubule formation, preventing efficient viral intercellular spread in the leafhopper *in vitro* and *in vivo* and subsequent transmission by the vector, without significant effect on viral multiplication in leafhopper cells.

## Introduction

Numerous plant viruses that cause serious losses to agricultural production are transmitted by vector insects, classified according to the type of transmission: nonpersistent, semipersistent and persistent [1]. Viruses transmitted in a persistent manner are further separated into two groups: propagative and nonpropagative [1]. In persistent-propagative transmission, viruses multiply in the vector insect during a latent period and can be transmitted to host plants until the death of the insects. Therefore, detailed analyses of viral propagation in the vector insects in persistent-propagative transmission would help disclose a mechanism that may lead to new strategies to control the transmission of the viruses by vector insects.

Plant viruses such as tospoviruses, tenuiviruses, plant rhabdoviruses and plant reoviruses, are transmitted by their respective insect vectors in a persistent-propagative manner, and thus they are designated as persistent-propagative plant viruses [1]–[4]. After their ingestion by insects during feeding on diseased plants, these viruses must enter and replicate in the epithelial cells of the alimentary canal of vector insects, then exit and move to the salivary glands to be transmitted to healthy plants [1]–[2]. While the replication sites and tissue tropism of these plant viruses in their respective insect vectors have been intensively studied, much less is known about how they spread from initially infected cells to adjacent cells or organs. Acquiring a better understanding of the intercellular spread of plant viruses in insects would lead to better strategies to disrupt the efficient transmission of plant viruses by insect vectors.

**Figure 1:**
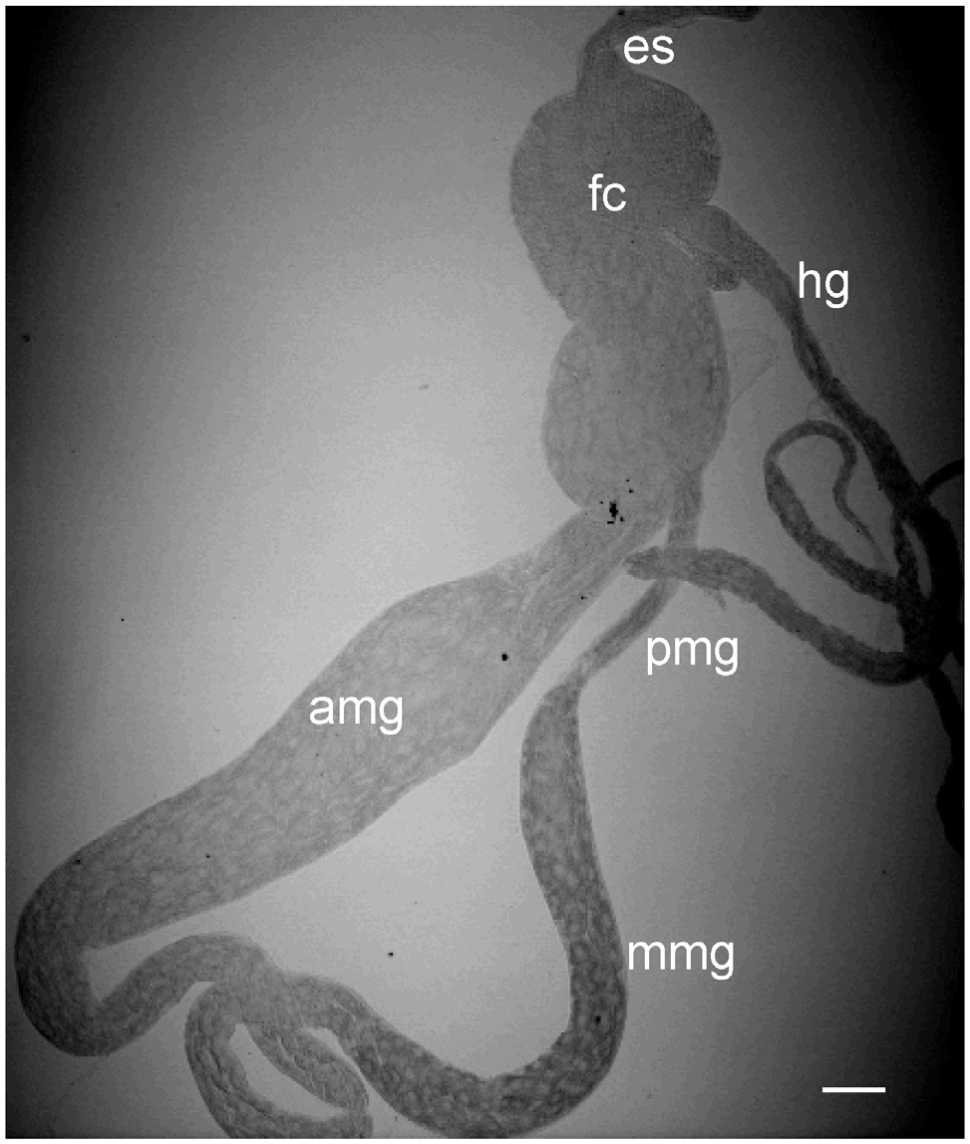
Transmitted light micrograph of partially dissected alimentary canal of leafhopper vector *N. cincticeps*. The alimentary canal of leafhopper consists of the esophagus (es), filter chamber (fc), anterior midgut (amg), middle midgut (mmg), posterior midgut (pmg) and hindgut (hg). Bar, 100 µm.

**Figure 2:**
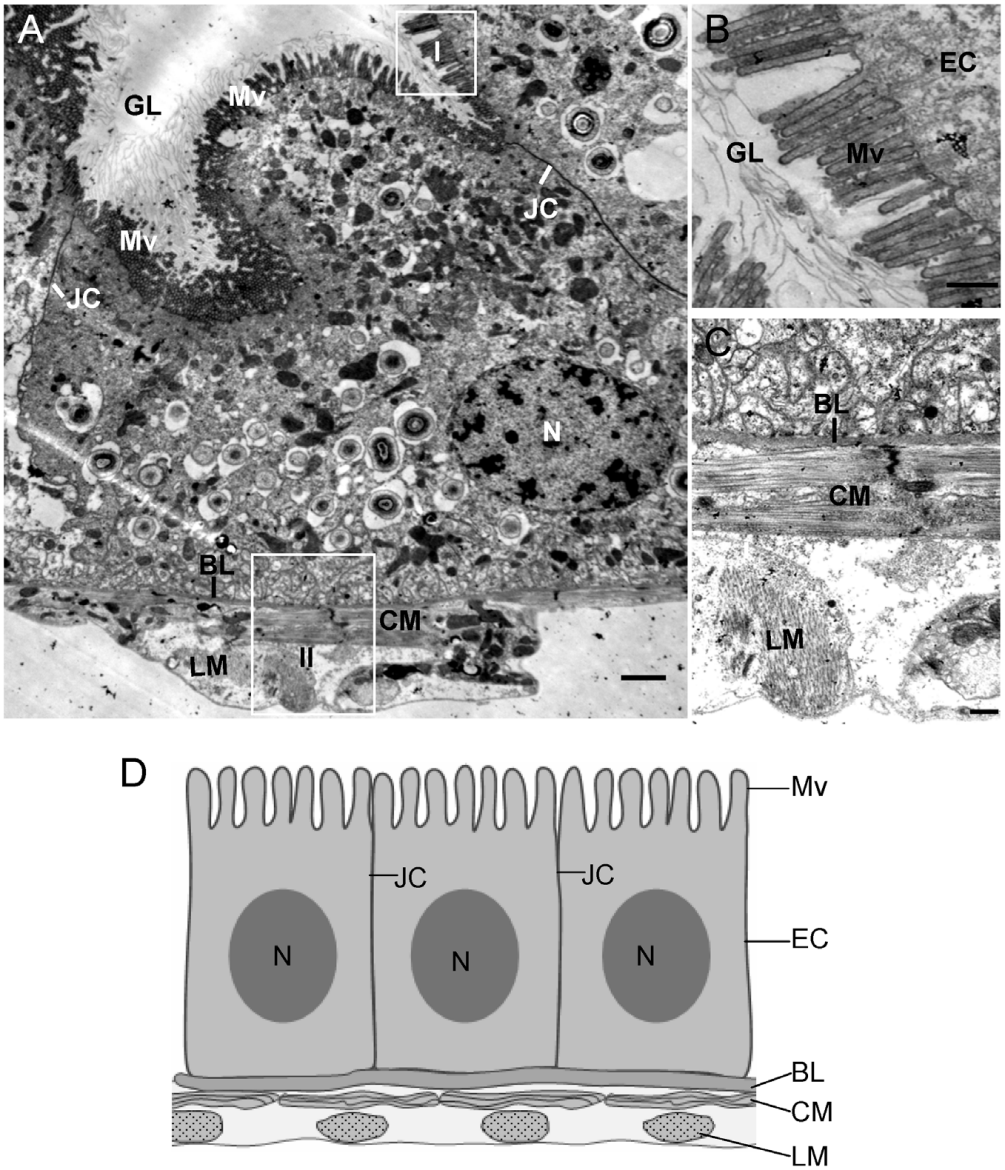
Transmission electron micrographs of the posterior midgut of leafhopper vector *N. cincticeps*. (**A**) Midgut epithelium, showing microvilli on lumen side and a basal lamina covered with muscle fibers. Panels **B** and **C** are enlarged images of the boxed areas I and II in panel A to show the microvilli and the visceral muscle tissues, respectively. The visceral muscle tissues consist of external longitudinal and internal circular muscle fibers. (**D**) Diagram of midugt epithelium. BL, basal lamina. CM, circular muscle. EC, epithelial cell. GL, gut lumen. LM, longitudinal muscle. Mv, microvilli. N, nucleus. JC, junctional complex. Bars, 2 µm (**A**) and 500 nm (**B–C**).

*Rice dwarf virus* (RDV), a member of the genus *Phytoreovirus* in the family *Reoviridae* [5], is transmitted by the green rice leafhopper *Nephotettix cincticeps* in a persistent-propagative manner and is transovarially transmitted [5]. The alimentary canal of leafhoppers consists of the esophagus, filter chamber, midgut and hindgut (Figure 1). The midgut is further divided into the anterior, middle and posterior regions (Figure 1). As in other types of leafhoppers [6]–[9], the alimentary canal of the leafhopper *N. cincticeps* is composed of a single layer of epithelial cells, with microvilli on the lumen side and basal lamina on the outer side, covered with muscle fibers (Figure 2). In epithelial tissues, cells are joined by intercellular junctional complexes (Figure 2), which may act as the physical barrier to prevent infection by microbes [10]. RDV encounters multiple membrane barriers in its path from the alimentary canal to the salivary glands in leafhopper vectors [9]. After ingestion of RDV particles by leafhoppers, virions first accumulate in the epithelial cells of the filter chamber, suggesting that the microvillar membrane of the filter chamber may contain cellular receptors for viral attachment and entry. Thus, the microvillar membrane of the filter chamber may form the first membrane barrier for successful infection of RDV in the body of leafhopper. RDV spreads to adjacent cells or organs such as the anterior midgut following the initial replication and accumulation of viruses in the epithelial cells of the filter chamber [9]. The molecular mechanisms by which RDV spreads from cell to cell among epithelial tissues are poorly understood. These cells or organs presumably constitute the second membrane barrier that must be penetrated by RDV to spread further in its insect host. Infection of the epithelial cells is followed by virus invasion of the visceral muscles lining the anterior midgut [9]. This observation suggests that RDV crossed the basement membrane of the anterior midgut, the third membrane barrier encountered by the virus, to infect the visceral muscles, possibly through the nerves associated with the muscle tissues [9]. The molecular mechanisms of cell-to-cell spread of RDV among the visceral muscle cells lining the midgut are unknown, but RDV might directly disseminate from the midgut-associated visceral muscles into the hemolymph, then into the salivary gland [9].

Studies of RDV–leafhopper interactions have provided evidence that the minor outer-capsid protein P2 and nonstructural protein Pns10 are required for vector transmissibility [11]–[16]. Nonsense mutations in the P2 or Pns10 genes abolish transmission of the virus by insects [16]. However, the absence of expression of these two proteins does not compromise the ability of the virus to replicate in plants [16]. The leafhopper vector cells in monolayers (VCMs) and the intact vector insects exhibited similar productive noncytopathic response to RDV infection [11]–[16]. Using VCMs, our previous studies indicated that RDV entered leafhopper cells through receptor-mediated, clathrin-dependent endocytosis using P2 as the viral attachment molecules [11]–[12]. Furthermore, RDV had been proposed to exploit tubular structures composed of the nonstructural protein Pns10 (Pns10 tubules) to move along actin-based filopodia extending toward neighboring cells, thus enhancing intercellular viral propagation among cultured leafhopper cells [14]–[15]. Previously, such tubules, containing RDV particles, were observed with electron microscopy in association with actin-based microvilli and seemed to pass through the microvilli of epithelial cells of the midgut in viruliferous leafhoppers [17]. Therefore, a defect in this machinery might be associated with the inability of mutant RDV to be transmitted by insect vectors. All these reports suggest that RDV might have evolved novel strategies to exploit virus-containing Pns10 tubules to cross through the epithelial cell of the alimentary canal in vector insects so that it can spread intercellularly.

In the present study, by combining an immunofluorescence technique and a feeding-based RNA interference (RNAi) technique to target Pns10 genes, we determined that RDV has evolved novel strategies to use virus-containing Pns10 tubules to move along actin-based microvilli of the epithelial cells and muscle fibers of visceral muscle tissues to facilitate intercellular spread in the intact insect vector.

## Results

### Pns10 tubules cross actin-based microvilli of the epithelial cell in viruliferous leafhoppers

**Figure 3:**
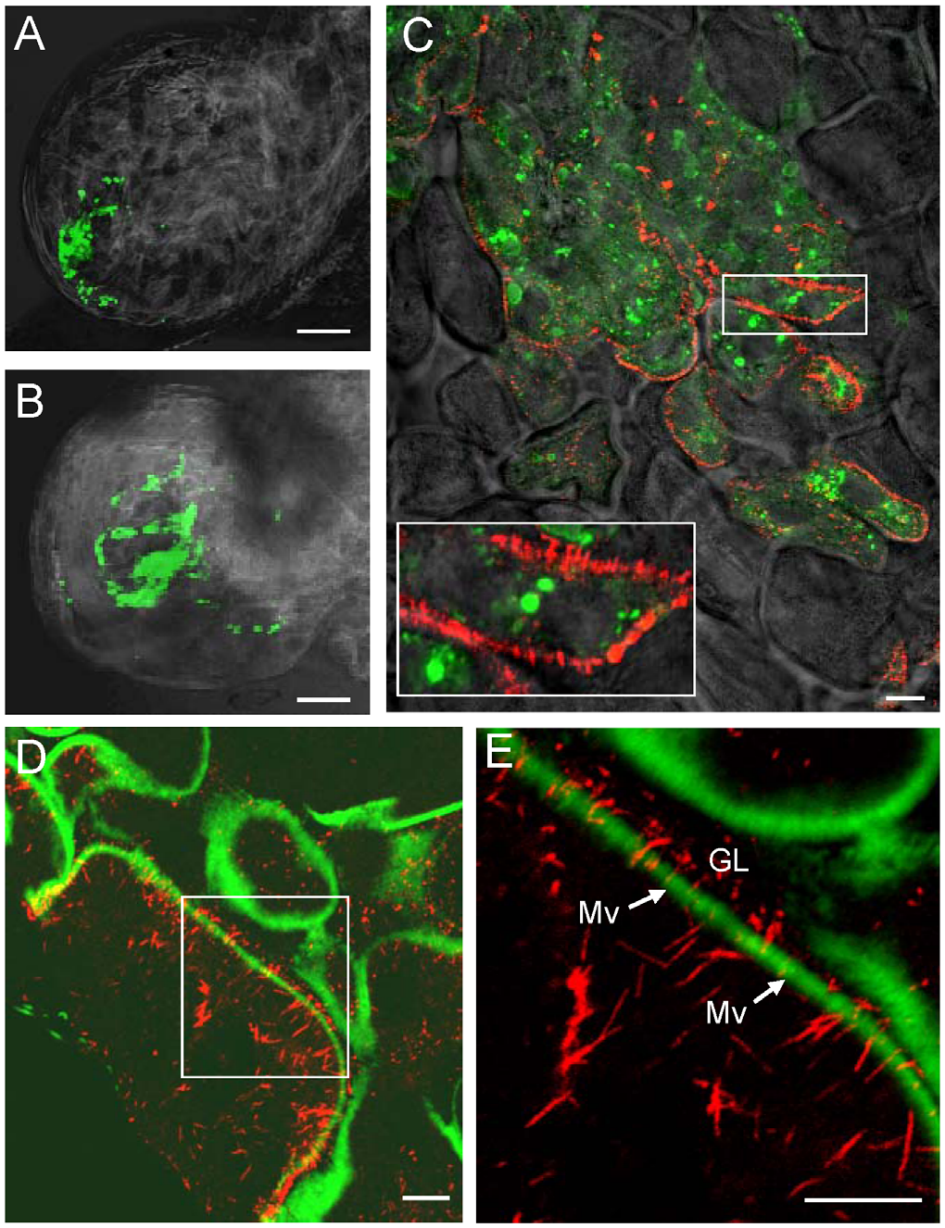
Pns10 tubules on actin-based microvilli of filter chamber in viruliferous leafhoppers. At 2-day (**A**) or 3-day (**B–E**) padp, leafhopper organs were immunolabeled for Pns10 tubules with Pns10-rhodamine (red), for RDV virions with virus-FITC (green), and for actin-based microvilli with FITC-phalloidin (green), then examined by confocal microscopy. (**A, B**) Fluorescence micrograph of filter chamber showing green fluorescence (virus antigens) with background visualized by transmitted light. (**C**) Image of filter chamber merged with images with green fluorescence (virus antigens), red fluorescence (Pns10 tubules) and background visualized by transmitted light. Inset indicates an enlarged image of the boxed area. (**D**) Image of filter chamber merged with image of green fluorescence (actin) and of red fluorescence (Pns10 tubules). (**E**) Enlarged image of boxed area in panel **D**. GL, gut lumen. Mv, microvilli. Images are representative of multiple experiments with multiple preparations. Bars, 350 µm (**A–B**) and 10 µm (**C–E**).

To study the development and distribution of Pns10 tubules in viruliferous leafhoppers over time, we used immunofluorescence microscopy to visualize the tubules in the epithelial cells of the alimentary canal during infection by RDV. At 2-day or 3-day post-first access to diseased plants (padp), internal organs dissected from leafhoppers were immunolabeled with Pns10-specific IgG conjugated to rhodamine (Pns10-rhodamine) and viral particle-specific IgG conjugated to FITC (virus-FITC), and then examined by fluorescence microscopy. At 2-day padp, small infection foci of virus antigens immunolabeled with virus-FITC were observed in a particular corner of the filter chamber (Figure 3A). At 3-day padp, the number of infected cells increased, forming larger infection foci (Figure 3B). Pns10 tubules were distributed at the periphery of infected epithelial cells and protruded from the cell surface, even at the edge of infection foci (Figure 3C). All these results suggested that RDV might spread from the initially infected epithelial cells to adjacent cells to form infection foci and that Pns10 might be responsible for the extension of tubules from the epithelial cell surface.

In the alimentary canal of insects, bundles of parallel actin filaments form the core of a microvillus [18]–[19]. Our previous data showed that Pns10 tubules could traffic along actin-based filopodia in VCMs through a specific but relatively low-affinity interaction between Pns10 and actin [14]. These data suggested that Pns10 tubules might pass through the bundles of actin filaments of microvilli. To examine this possibility, we dissected internal organs from leafhoppers at 3-day padp and immunolabeled them with Pns10-rhodamine and the actin dye FITC-phalloidin. This double labeling revealed that actin-based microvilli along the surface of the epithelial cells of the filter chamber were filled with Pns10 tubules (Figure 3D, E), suggesting that Pns10 tubules could pass through the actin-based microvilli from inside of infected cells into the lumen of the filter chamber.

**Figure 4:**
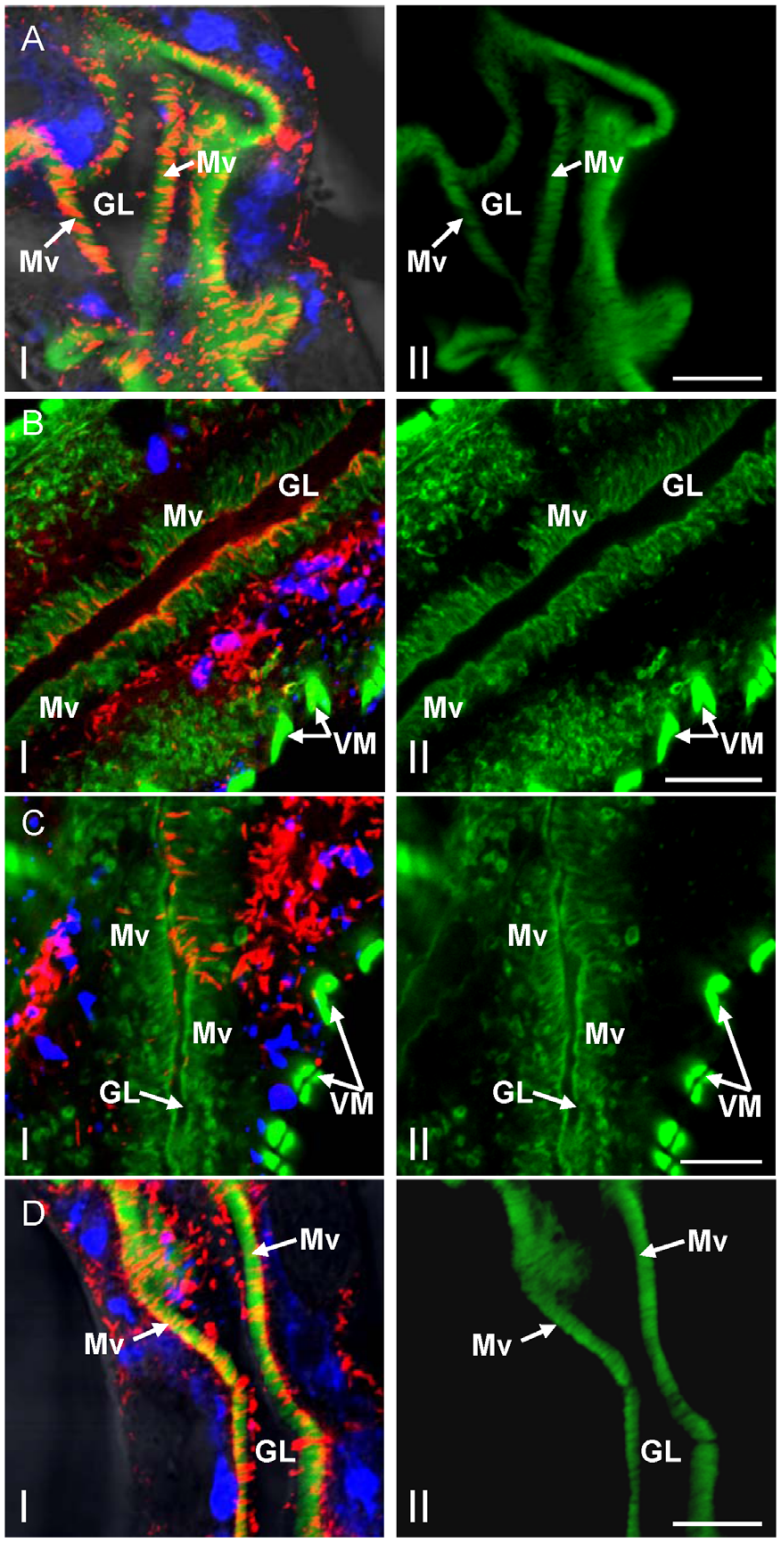
Pns10 tubules on actin-based microvilli of midgut in viruliferous leafhoppers. At 6-day padp, leafhopper organs were immunolabeled for Pns10 tubules with Pns10-rhodamine (red), for RDV virions with virus-Alexa Fluor 647 (blue), and for actin-based microvilli with FITC-phalloidin (green), then examined with confocal microscopy. Images of anterior midgut (**A**), middle midgut (**B, C**) and posterior midgut (**D**) are shown. Images I in **B** and **C** were merged with red fluorescence (Pns10 tubules), blue fluorescence (virus antigens) and green fluorescence (actin). Images I in **A** and **D** were merged with red fluorescence (Pns10 tubules), blue fluorescence (virus antigens) and green fluorescence (actin) with background visualized by transmitted light. Images II were single green fluorescence (actin) to show the actin-based microvilli. GL, gut lumen. Mv, microvilli. VM, visceral muscles. Images are representative of multiple experiments with multiple preparations. Bars, 10 µm.

After replication and accumulation of progeny virions in the epithelial cells of filter chamber, RDV virions exit and move into the adjacent midgut [9]. To determine whether virus-containing Pns10 tubules can pass through the microvilli of the midgut, at 6-day padp, internal organs dissected from leafhoppers were immunolabeled with Pns10-rhodamine, viral particles-specific IgG conjugated to Alexa Fluor 647 carboxylic acid (virus-Alexa Fluor 647) and actin dye FITC-phalloidin 6-day padp. In the anterior midgut, abundant Pns10 tubules extended from or passed through the actin-based microvilli of infected epithelial cells (Figure 4A). In the middle and posterior midgut, abundant Pns10 tubules extended from the actin-based microvilli along the surface of infected epithelial cells of the midgut (Figure 4B–D), consistent with previous electron microscopy observations that tubules containing RDV particles were associated with the microvilli of the midgut in viruliferous leafhoppers [17]. A few Pns10 tubules had successfully crossed into the gut lumen (Figure 4B–D). Furthermore, some Pns10 tubules were associated with the actin-based microvilli of neighboring cells (Figure 4C), suggesting that RDV might exploit these tubules to facilitate viral cell-to-cell spread among epithelial cells in the midgut of the leafhopper.

**Figure 5:**
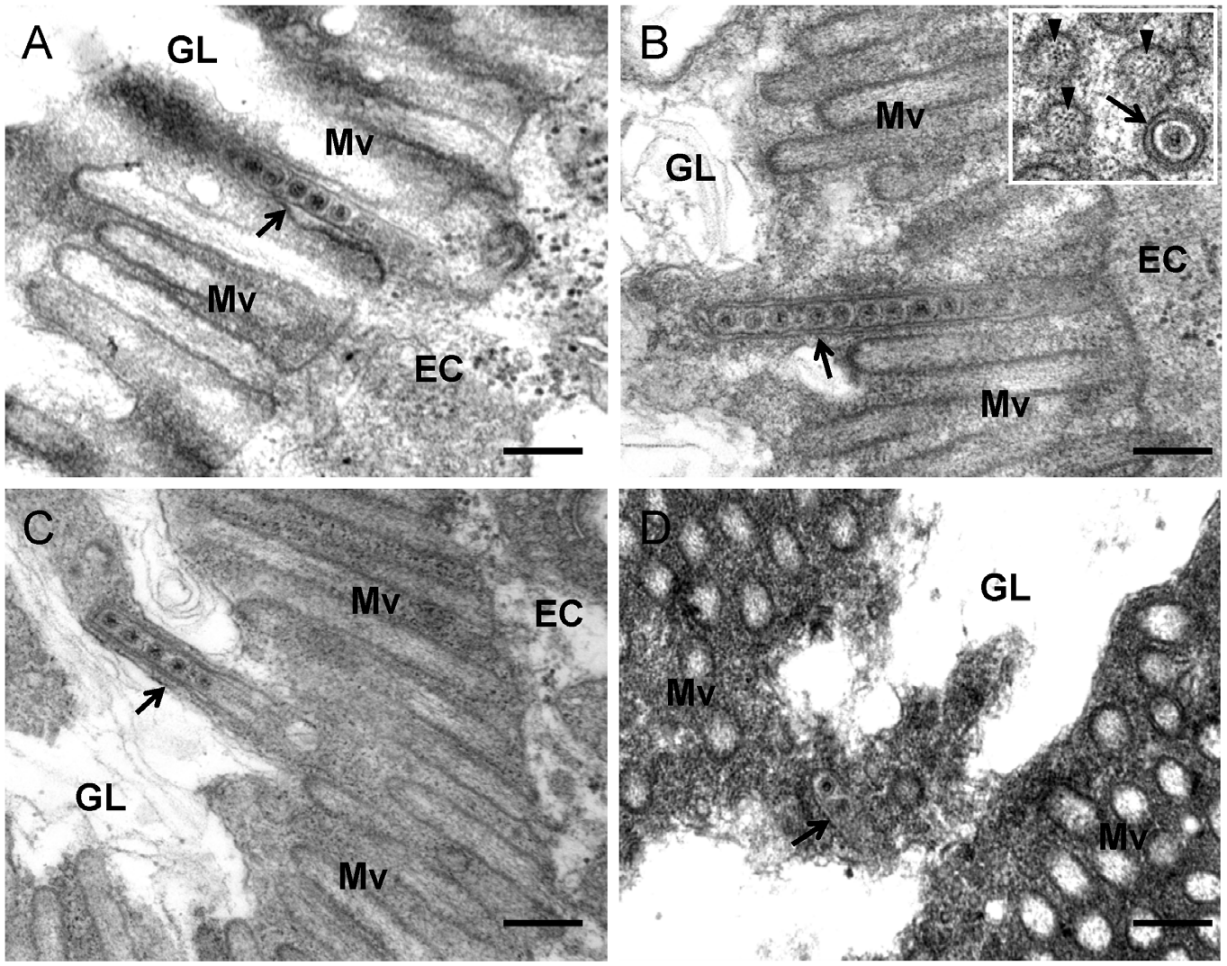
Transmission electron micrographs showing the association of virus-containing tubules with microvilli of anterior midgut in viruliferous leafhoppers. (**A**) Closed-end of tubule (arrow) inserted into a microvillus. (**B**) Closed-end tubule (arrow) in contact with the inner side of distal end of microvillus. Inset, transverse section of microvillus of about 100 nm in diameter, with a virus-containing tubule (arrow) inside. Arrowheads indicate actin filaments within the microvillus. (**C**) Elongated tubule-associated microvillus (arrow) has formed membrane protrusion toward the lumen. (**D**) Tubule (arrow) in the lumen. EC, epithelial cell. GL, gut lumen. Mv, microvilli. Bars, 200 nm.

To confirm our observations, we further used electron microscopy to visualize the tubules in the epithelial cells of the alimentary canal during infection by RDV. The alimentary canal of leafhopper is formed by epithelial cells that lie on basal lamina surrounded by external longitudinal and internal circular muscle fibers (Figure 2). Numerous brush border microvilli on the surface of epithelial cells extend into the lumen (Figures 2B, 5, S1). RDV infection induced the formation of tubules approximately 85 nm in diameter, with viral particles inside, in the epithelial cells of the filter chamber and midgut (Figures 5, S1). These tubules appeared to be passing through the microvilli from inside the infected cells into the lumen (Figures 5, S1), consistent with the observations with immunofluorescence microscopy (Figure 4).

A careful analysis of electron micrographs revealed the possible process of the crossing of tubules through the microvilli. The closed-end of the tubules first was enclosed inside the microvillus (Figure 5A), and then entire tubules extended into the microvillus (Figure 5B). We observed that the closed-end of the tubule made contact with the inner side of the distal end of the microvillus (Figure 5B), which may drive the elongation of the microvillus to form a membrane protrusion towards the lumen (Figure 5C). Finally, the tubules were released in the lumen from these microvilli (Figure 5D). Free virions were absent in the microvilli (Figures 5, S1). Our previous electron tomographic microscopy indicated that RDV particles were fixed on the inner surface of the tubules; thus they did not freely diffuse within the tubules [20]. All these observations indicated that RDV particles were accompanied by Pns10 tubules to pass though the microvilli into the lumen.

### Pns10 tubules colocalize with actin-based muscle fibers of visceral muscle tissues surrounding the midgut of viruliferous leafhoppers

**Figure 6:**
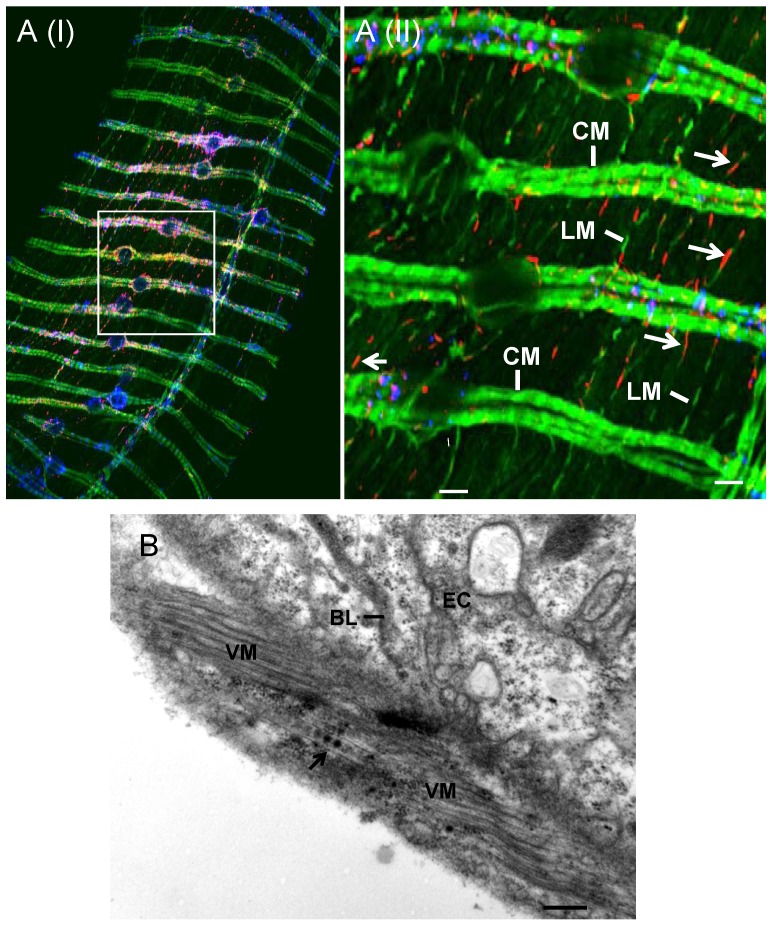
Pns10 tubules localized along actin-based muscle fibers that surround the anterior midgut in viruliferous leafhopper. (**A**) Confocal micrograph showing the association of Pns10 tubules with actin-based longitudinal muscle fibers. At 12-day padp, leafhopper organs were immunolabeled for Pns10 tubules with Pns10-rhodamine (red), for RDV virions with virus-Alexa Fluor 647 (blue), and for actin-based microvilli with FITC-phalloidin (green), then examined by confocal microscopy. Images show the blue fluorescence (virus antigens) and red fluorescence (Pns10 tubules) overlapped with the green fluorescence (actin). Image II is a higher magnification view of boxed area in image I to show that Pns10 tubules (red; arrows), rather than virus antigens (blue), co-localize with actin-based longitudinal muscle fibers (green) that seem to bridge two adjacent circular muscle cells. Images are representative of multiple experiments with multiple preparations. (**B**) Transmission electron micrograph showing the association of virus-containing tubule (arrow) with visceral muscle tissues lining anterior midgut. BL, basal lamina. CM, circular muscle. EC, epithelial cell. LM, longitudinal muscle. VM, visceral muscle. Bars, 20 µm (image I in **A**), 80 µm (image II in **A**) and 200 nm (**B**).

Our previous observations indicated that infection of RDV in the epithelial cells was followed by virus invasion of the visceral muscle tissues lining the anterior midgut [9]. To determine whether Pns10 tubules were associated with the visceral muscle lining the anterior midgut, at 12-day padp, we dissected the internal organs from leafhoppers and immunolabeled them with Pns10-rhodamine, virus-Alexa Fluor 647 and FITC-phalloidin. As shown in Figure 6A, the visceral muscles formed by actin-based longitudinal muscle fibers and circular muscle fibers were visible as a fluorescent lattice pattern. Short Pns10 tubules were specifically associated with single actin-based longitudinal muscle fiber that connected with circular muscle fiber bundles (Figure 6A). In contrast, free virions were absent in these longitudinal muscle fibers (Figure 6A). Electron microscopic observations confirmed that virus-containing tubules were associated with the visceral muscle tissues lining the anterior midgut during infection by RDV (Figure 6B). These observations implied that RDV might exploit Pns10 tubules to traffic along actin-based muscle fibers to facilitate the lateral spread of RDV.

### RNAi induced by dsPns10 inhibits the assembly of Pns10 tubules and secondary infection of neighboring cells by RDV in VCMs

**Figure 7:**
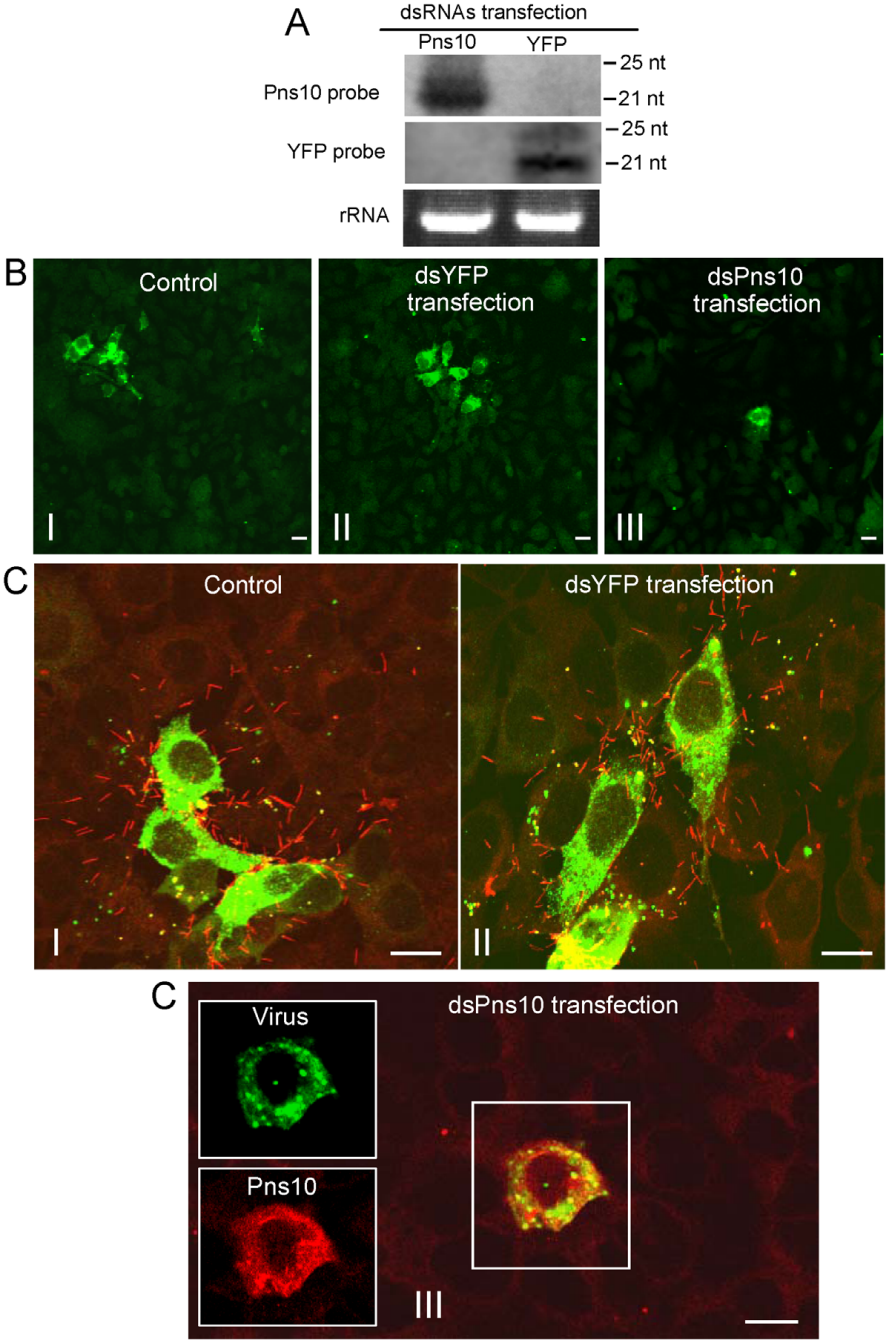
RNAi induced by dsPns10 inhibiting the assembly of Pns10 tubule and secondary infection of neighboring cells by RDV in VCMs. (**A**) Detection of siRNAs in VCMs transfected with synthesized dsPns10 or dsYFP at 72 h after transfection. Approximately 5 µg of total RNA was probed with DIG-labeled transfected RNA. Lower panel: detection of 5.8S rRNA as a control to confirm loading of equal amounts of RNA in each lane. (**B, C**) Intercellular spread of RDV was inhibited by RNAi induced by dsPns10 in VCMs. At 24 h after transfection with cellfection transfection reagent (control; images I), dsYFP (images II) or dsPns10 (images III), VCMs were inoculated with RDV at a low MOI (0.001) and cultured in the presence of virus-neutralizing antibodies, which prevent free virus infection. At 3 days p.i., cells were immunolabeled for RDV virions with virus-FITC (green) or for Pns10 tubules with Pns10-rhodamine (red), and then examined by confocal microscopy. Images in **B** show green fluorescence from virus antigens in widefield view. Images in C are composites of those for green fluorescence (virus antigens) and red fluorescence (Pns10 tubules). Insets in image III of panel **C** show green fluorescence (virus antigens) and red fluorescence (Pns10 antigens) of the merged images in the boxed area. Images are representative of multiple experiments with multiple preparations. Bars, 20 µm.

We initially used an *in vitro* system of VCMs to examine RNAi activity caused by dsRNA specific for Pns10 gene. VCMs on a coverslip were transfected with dsRNAs specific for Pns10 gene of RDV (dsPns10) or segment encoding for yellow fluorescence protein (YFP) (dsYFP) via Cellfection-based transfection. Viability tests showed no obvious differences among transfected cells, demonstrating absence of toxicity of the transfection reagent and dsRNAs to VCMs (data not shown). RNAi is demonstrate by the appearance of small interfering RNAs (siRNAs) corresponding to an mRNA target sequence [21]. Therefore, the presence of siRNAs specific for Pns10 or YFP genes was analyzed by northern blots at 72 h after transfection of synthesized dsRNAs. Small RNAs approximately 21 nt long were detected from RNAs extracted from dsPns10- or dsYFP-transfected cells (Figure 7A), showing that RNAi was induced in the VCMs after transfection with dsRNAs. Then, 24 h after transfection, the VCMs were inoculated with RDV at a low MOI of 0.001 and then cultured in the presence of virus-neutralizing antibodies. At this low MOI, viral infection rate was below 1%, and secondary infection was clearly visible, as described previously [14], [15]. Cells were fixed 3 days post-inoculation (p.i.) and immunolabeled with virus-FITC and Pns10-rhodamine. In VCMs transfected with dsYFP or treated with Cellfection alone (i.e., control), small foci of 4 to 10 infected cells were visible in the presence of virus-neutralizing antibodies, consistent with the spread of RDV from an initially infected cell to adjacent cells (Figure 7B, C, panels I and II). Furthermore, abundant Pns10 tubules protruded from such infected cell surfaces and were scattered outside the cells (Figure 7C, panels I, II). On the other hand, in VCMs transfected with dsPns10, RDV was restricted to the initially infected cells in the presence of virus-neutralizing antibodies (Figure 7B, C, panels III). Furthermore, the formation of Pns10 tubules had been blocked (Figure 7C, panel III), indicating that the expression of Pns10 had been knocked down by RNAi induced by dsPns10.

**Figure 8:**
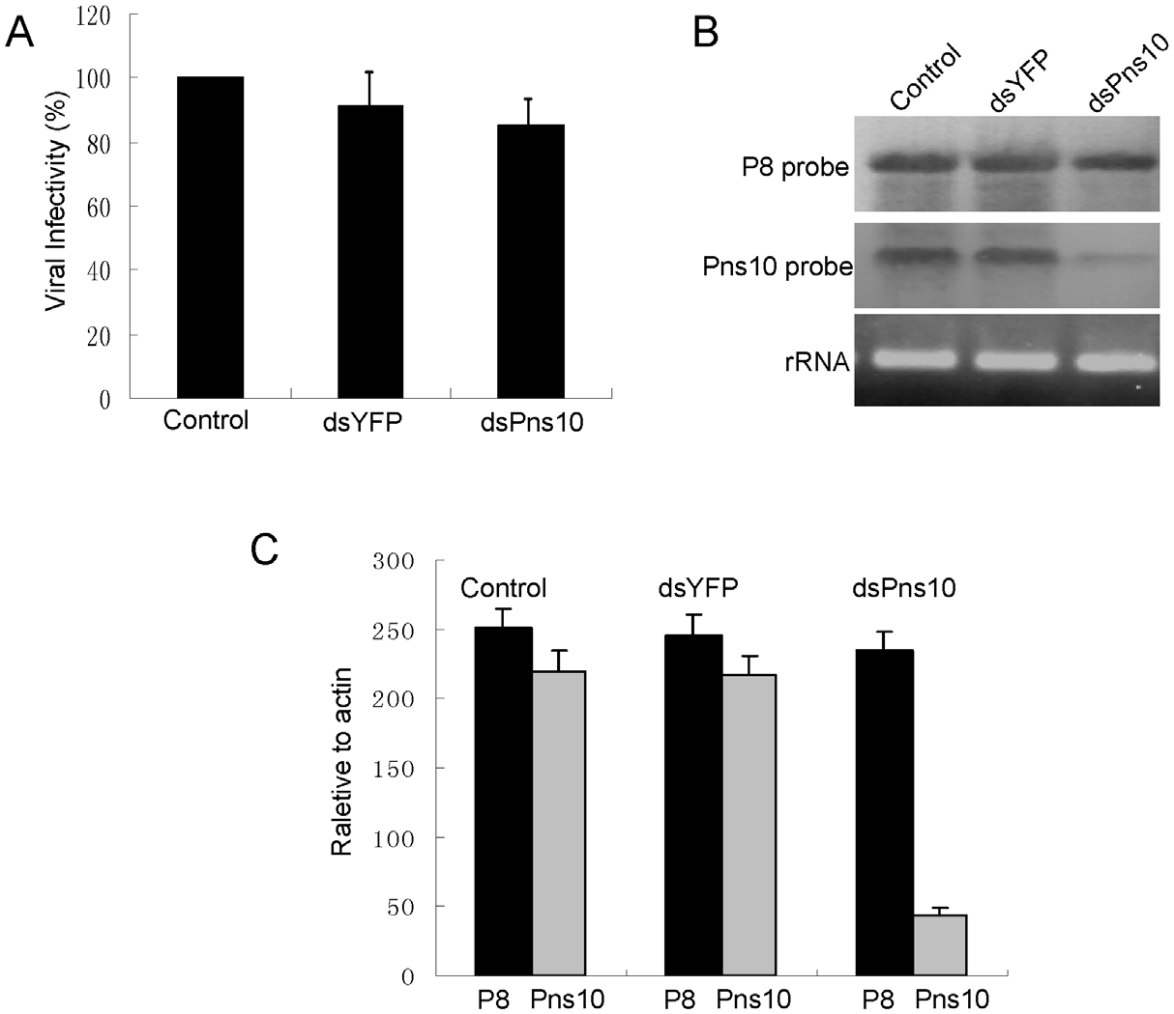
RNAi induced by dsPns10 knockdown the expression of Pns10 without significantly inhibiting virus multiplication in VCMs. (**A**) Effects of the treatment of dsRNAs on multiplication of cell-associated RDV in VCMs. Viral titers were determined in duplicate by the fluorescent focus assay (see text for details). Error bars indicate standard deviations from three independent experiments. (**B**) Transfection of dsPns10 in VCMs results in a significant reduction in level of plus-strand RNA of Pns10 gene, without greatly inhibiting synthesis of plus-strand RNA of P8 gene, as revealed by northern blot. VCMs were transfected with transfection reagent (control), dsYFP or dsPns10, inoculated with RDV at an MOI of 10, then harvested 72 h later. Approximately 5 µg of total RNAs were probed with DIG-labeled negative-sense RNA transcripts of Pns10 or P8 genes. Lower panel: detection of 5.8S rRNA as a control to confirm loading of equal amounts of RNA in each lane. Image is representative of multiple experiments with multiple preparations. (**C**) Transfection of dsPns10 in VCMs caused about 80% reduction in level of plus-strand RNA of Pns10 gene, without greatly inhibiting synthesis of plus-strand RNA of P8 gene, as revealed by RT-qPCR assay. VCMs were transfected with transfection reagent (control), dsYFP or dsPns10, inoculated with RDV at an MOI of 10, then harvested 72 h later. Approximately 5 µg of total RNAs was extracted with TRIzol Reagent. The results of RT-qPCRs were normalized to the level of leafhopper actin gene. Error bars indicate standard deviations from three independent PCRs.

Our previous findings indicated that the blockage of Pns10 tubule formation by chemical inhibitors did not significantly affect viral multiplication in VCMs [14]–[15]. To further examine whether the treatment with dsPns10 had any effects on the multiplication of RDV in VCMs, 24 h after transfection with dsRNAs, VCMs were inoculated with RDV at an MOI of 10 to guarantee viral infection rate was 100%, and then cell lysates were collected at 72 h. As shown in Figure 8A, dsPns10 reduced the titer of cell-associated viruses by 7–11%, suggesting that RNAi induced by dsPns10 had no substantial effect on the accumulation of RDV in VCMs.

The effects of RNAi induced by dsPns10 on the synthesis of viral plus-strand RNAs were analyzed with Northern blots. As shown in Figure 8B, transfection with dsPns10 resulted in a marked reduction of the synthesis of plus-strand RNA of Pns10 gene, but without significant effect on the synthesis of plus-strand RNA of viral major outer capsid protein P8 gene. The effects of RNAi induced by dsPns10 on the synthesis of viral plus-strand RNAs were further confirmed by quantitative real-time RT-PCR (RT-qPCR) assay. RT-qPCR assay showed that the treatment of dsPns10 caused about 80% reduction in the level of plus-strand RNA of Pns10 gene, relative to a constant amount of plus-strand RNA of P8 gene (Figure 8C). Thus, RNAi induced by dsPns10 could specifically knock down the expression of Pns10 gene, but caused little reduction of the expression of other viral genes, a finding consistent with its failure to effectively inhibit viral multiplication in VCMs. All these results suggested that the inhibition of the assembly of Pns10 tubules, due to RNAi induced by dsPns10 resulted in the failure of Pns10 tubules to protrude beyond the cell surface and in the subsequent lack of infection of neighboring cells by RDV.

### Ingestion of dsPns10 knocks down expression of Pns10 protein and slows RDV spread in the intact insect

**Figure 9:**
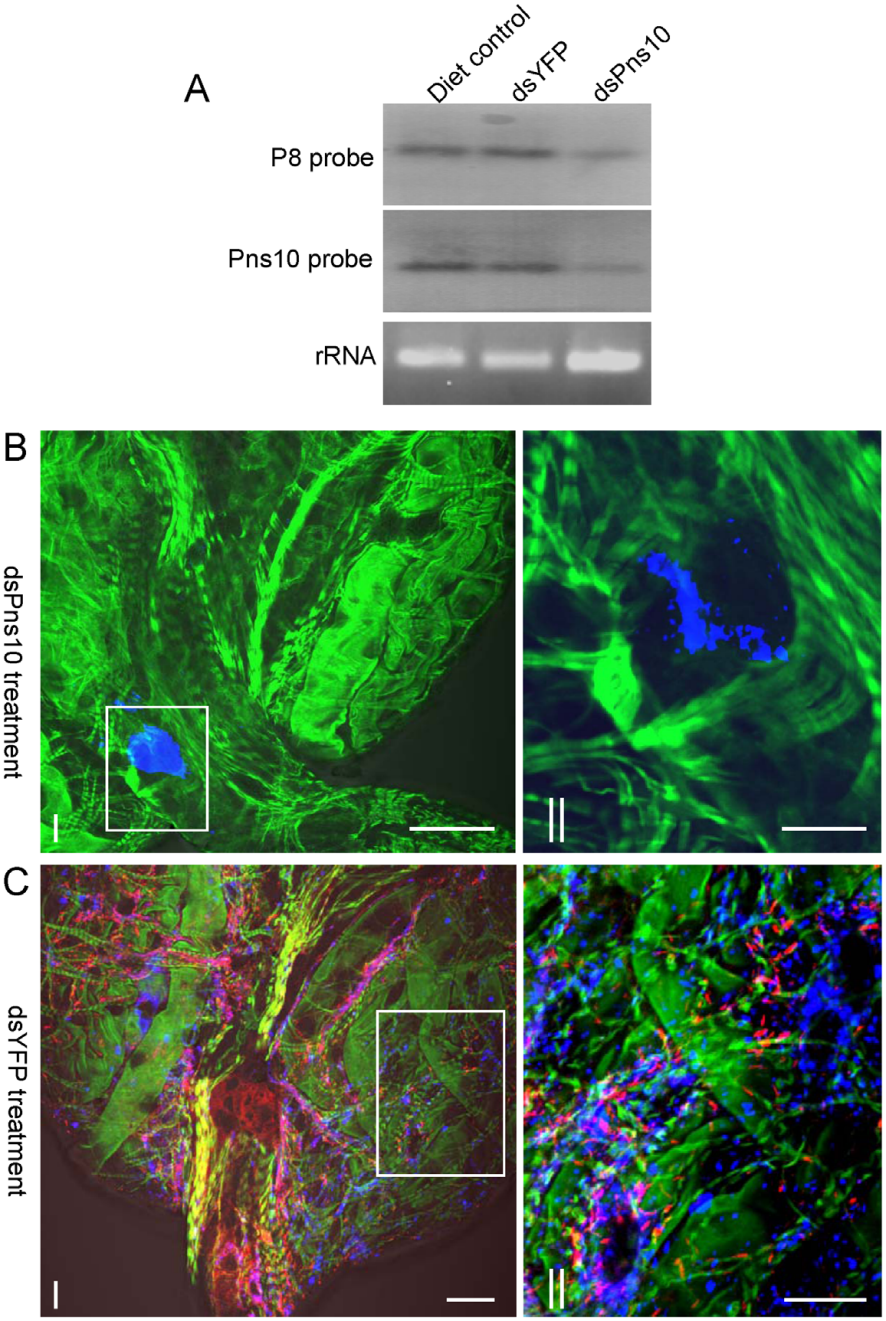
Ingestion of dsPns10 knocks down expression of Pns10 proteins and slows RDV spread in the intact insect. (**A**) RNAi induced by dsPns10 reduced the synthesis of viral plus-strand RNAs from Pns10 and P8 genes in leafhoppers. Second-instar nymphs of leafhopper were fed with dsRNAs or sucrose diet (control) via membrane-feeding. At 12-day padp, approximately 5 µg of total RNAs extracted from leafhoppers organs were probed with DIG-labeled negative-sense RNA transcripts of Pns10 or P8 genes. Lower panel: detection of 5.8S rRNA as a control to confirm loading of equal amounts of RNA in each lane. Image is representative of multiple experiments with multiple preparations. (**B, C**) Ingestion of dsPns10 via membrane-feeding blocking the formation of Pns10 tubules and RDV spread in the filter chamber of leafhopper vectors. The second-instar nymphs of leafhopper were fed with dsPns10 (**B**) or dsYFP (**C**) via membrane-feeding. At 12-day padp, leafhopper organs were immunolabeled for Pns10 tubules with Pns10-rhodamine (red), immunolabeled for RDV virions with virus-Alexa Fluor 647 (blue), and immunolabeled for actin with FITC-phalloidin (green). Images II are higher magnification views of boxed areas in images I in panels **A** and **B**, respectively, to show that the assembly of Pns10 tubules (red) and the spread of RDV (blue) was blocked in the filter chamber of leafhoppers that received dsPns10. Images are representative of multiple experiments with multiple preparations. Bars, 100 µm (images I in **A** and **B**), 350 µm (images II in **A** and **B**).

To address the potential function of Pns10 in viral transmission by insect vectors, we tested whether RNAi induced by ingestion of dsPns10 could inhibit viral infection in intact leafhoppers. In preliminary experiments, ingestion of dsRNA by the leafhoppers resulted in no phenotypic abnormalities (data not shown). The percentage of viruliferous insects after ingestion of dsRNAs was analyzed by RT-PCR of the genes for the nonstructural protein Pns10 and the major outer capsid protein P8 of virus. When one of the genes was detected, the other gene was also detected in all cases. At 12-day padp, about 55% (*n* = 100, 3 repetitions) of leafhoppers that received dsYFP had the Pns10 and P8 genes (Table 1). In contrast, about 12% (*n* = 100, 3 repetitions) of leafhoppers that received dsPns10 had the Pns10 and P8 genes (Table 1). The number of positive samples did not differ significantly between leafhoppers that received dsYFP and a sucrose diet control (Table 1). The effect of dsRNA treatment on the synthesis of viral plus-strand RNAs from the Pns10 and P8 genes was further analyzed with Northern blots. In agreement with the RT-PCR results, accumulation of viral plus-strand RNAs of the Pns10 and P8 genes was greatly reduced in RNA fractions isolated from leafhoppers receiving dsPns10 as compared with leafhoppers receiving dsYFP or the sucrose diet control (Figure 9A). These results confirmed that the expression of Pns10 could be knocked down by RNAi induced by synthesized dsRNA that were fed to the insects using a membrane-feeding method.

**Table 1:** Ingestion of dsPns10 via membrane feeding strongly inhibited viral spread and transmission by insect vectors.

Insects	No. of positive insects with Pns10 and P8 genes detected by RT-PCR at 12-day padp (*n* = 100)				No. of positive insects with virus antigens and Pns10 tubules in different tissues at 12-day padp^a^ (*n* = 30)				No. of positive insects that transmitted viruses to rice seedlings at 22-day padp (*n* = 60)		
	I	II	III	Exp. no.	fc^b^ (limited)	fc^b^ (extensive)	mg	sg	I	II	III
dsPns10	13	10	12	I	12	3	3	2	1	2	1
				II	11	5	5	2			
				III	9	6	5	3			
dsYFP	52	58	54	I	0	18	14	9	7	8	10
				II	0	17	17	11			
				III	0	15	15	7			
diet control	58	60	63	I	0	16	16	10	8	10	10
				II	0	17	13	11			
				III	0	16	12	8			

a ,The filter chamber (fc), midgut (mg) and salivary gland (sg) of leafhoppers were examined for immunofluorescence of virus antigens and Pns10 tubules.

b , Distribution of virus antigens and Pns10 tubules was assessed as either in a limited area or in an extensive area of filter chamber (fc).

To determine whether blocking the formation of virus-containing Pns10 tubules would inhibit viral spread among leafhopper tissues, we dissected the internal organs from leafhoppers at 12-day padp, and then immunolabeled them with Pns10-rhodamine, virus-Alexa Fluor 647 and actin dye FITC-phalloidin. In about 36% of leafhoppers that received dsPns10, virus antigens were restricted to a small area on the corner of the filter chamber, the initial entry site of RDV, and the formation of Pns10 tubules was mostly inhibited in these regions (Figure 9B, Table 1). By contrast, both viral antigens and Pns10 tubules were detected in the filter chamber, midgut and salivary glands in about 56% leafhoppers receiving dsYFP, but only in about 16% of those receiving dsPns10 (Figure 9C, Table 1), corresponding to the proportion of positive samples checked by RT-PCR, as shown already. All these results suggested that RNAi induced by ingestion of dsPns10 is activated in about 70% of RDV-positive leafhoppers, which may largely reflect limitations in uptake efficiency (about 70%) of dsPns10 into the intestine tissues of the leafhopper. Taken together, the formation of Pns10 tubules was heavily impaired due to the knockdown of Pns10 expression in leafhoppers that received dsPns10, which would lead to significant inhibition of the efficient spread of RDV in the body of the leafhoppers. As expected, the ingestion of dsPns10 by leafhoppers significantly suppressed vector transmission of the virus (Table 1).

## Discussion

RDV particles have been observed in tubules in microvilli in ultrathin sections of viruliferous vector insects [17]. This tubule, about 85 nm in diameter, is now regarded as the Pns10 tubule and plays an important role in viral spread and infection of neighboring uninfected cells [14], [20]. Recently, we studied RDV and its sequential infection of the internal organs of its vector insect over time by analyzing the spread of virus antigens after ingestion of the virus by the vector insect [9]. The virus first accumulated in epithelial cells of the filter chamber, progressed to the anterior midgut, and then spread to visceral muscles surrounding the anterior midgut [9]. In the present study, we focused on the distribution of Pns10 tubules in viruliferous vector insects over time and found that Pns10 tubules, with viral particles inside, crossed actin-based microvilli of epithelial cells (Figures 3–5), a finding for the first time in virus research, suggesting the passage of the virus through the microvillus membrane. Pns10 protein, with its ability to bind actin via a specific but relatively low-affinity interaction [14], may permit the Pns10 tubules to traffic along the actin-based microvilli of the epithelial cells of midgut. Results from electron microscopy revealed that virus-containing tubule extended along the actin filaments in the microvillus and finally arrived at the distal end of the microvillus (Figures 5, S1). The continuous contact of the tubule with the distal end of the microvillus apparently promoted the elongation of the microvillus to form a membrane protrusion towards the lumen (Figure 5), consistent with a mechanism underlying the outgrowth of cellular protrusions driven by actin-polymerization [22], [23]. The tubules seemed to be released from the broken microvilli in the lumen (Figure 5D). This scenario of RDV spread also occurred after the virus propagated in the visceral muscle tissues surrounding the anterior midgut, where lateral spread of the Pns10 tubules along actin-based muscle fiber might facilitate viral cell-to-cell movement through the muscle tissue (Figure 6). All these results suggest that movement of virus-containing Pns10 tubules along actin-based cellular protrusions would enable RDV to efficiently spread in various organs in viruliferous insects.

We previously provided indirect evidence that Pns10 tubule played an important role in RDV spread based on data showing that inhibition of the extension of actin-based cellular protrusion resulted in the failure of Pns10 tubule extension, which eventually incapacitated viral spread among insect vector cells [14]–[15]. To gain direct evidence that Pns10 tubule plays a critical role in the intercellular spread of virus in inset vector cells, here we used an RNAi technique to interrupt the formation of Pns10 tubules, then analyzed its effect on viral spread. VCM is an *in vitro* experimental system which enables us to clarify the molecular entities responsible for the biological events during viral replication [14]–[15]. In VCMs in the presence of neutralizing antibodies to avoid infection by free virions, virus antigens spread to several neighboring cells via Pns10 tubules (Figure 7B–C; Control and dsYFP transfection), as reported earlier [14]. In contrast, after the knockdown of Pns10 expression by RNAi induced by dsPns10, only one cell was infected even at 3 days after inoculation (Figure 7B–C, dsPns10 transfection). These results, together with the fact that dsPns10 specifically knocked down the expression of Pns10, but did not significantly affect viral multiplication (Figure 8) demonstrate that Pns10 tubule itself plays an important role in viral spread in VCMs. Because the use of a cultured monolayer system enables a synchronous and total cell response to treatment with dsRNAs, our RNAi system coupled with the use of VCMs should enable us to disclose the biological activities of each of the phytoreovirus-encoded proteins.

The number of RDV-positive insects detected by RT-PCR was significantly reduced in insect populations treated with dsPns10 as compared to those treated with dsYFP or diet control (Table 1). When analyzed in more detail, the knockdown of Pns10 expression did not interfere with the initial infection of virus into the filter chamber (Table 1, Figure 9B), the primary site for viral attachment and entry [9], corresponding with a previous hypothesis that RDV uses the minor capsid protein P2 as a viral attachment molecule to bind to the cellular receptor on the microvillar membrane of the filter chamber to enable viral uptake into the epithelial cells [9], [11]–[12]. In contrast, the knockdown of Pns10 expression strongly inhibited the extensive spread of RDV in the filter chamber and efficient intercellular spread of the virus to other organs such as the midgut and salivary gland in the body of its vector insects (Table 1). As a persistent-propagative plant virus, after moving into the epithelial cells of the filter chamber, RDV must replicate and assemble progeny virions, which would then spread to neighboring cells or other organs such as the midgut and salivary glands. Because the knockdown of Pns10 expression by treatment with dsPns10 or the blockage of Pns10 tubule formation by chemical inhibitors [15] did not significantly affect viral multiplication in leafhopper cells (Figure 8), we determined that the slow spread of RDV in the body of the dsPns10-treated insects was directly caused by a significant loss in the functioning of Pns10 tubules in viral intercellular spread from initially infected epithelial cells in the filter chamber. These results indicated that the interference of Pns10 tubules with viral spread in vector insect cells (Figure 7) significantly inhibited viral proliferation in intact vector insects, which would eventually significantly reduce viral transmissibility by the vector insects (Table 1). Results mentioned earlier, together with the direct evidence showing that Pns10 tubule is responsible for viral spread *in vitro* (Figure 7) and the observation that virus-containing Pns10 tubules are located along actin-based cellular machinery as they spread into various organs in the viruliferous insect (Figures 3–6), demonstrate that Pns10 tubules containing viral particles, play a critical role in viral spread in vector insects, enabling to accomplish a latent period for the virus, and subsequent ability to transmit the virus to plant hosts. Additional indirect evidence for the involvement of Pns10 tubules as viral determinants for virus transmission is that a Pns10-protein-deficient isolate of RDV failed to be transmitted by insect vectors [16]. However, free RDV particles may also exploit receptors on the intercellular junctional complex in the epithelial tissues to enable viral intercellular spread, as in the case of several animal viruses [24]; this issue should be further investigated. The novel model for viral intercellular spread presented here is thought to be advantageous over infection by a cell-free virus because the virus is protected from host immune responses.

Our results showing that RNAi was induced in leafhopper *N. cincticeps* by treatment with dsRNA targeting a viral gene, confirmed a recent finding that RNAi can be induced in leafhopper *Homalodisca vitripennis* (glassy winged sharpshooter) by treatment with dsRNA targeting an insect gene [25]–[26]. Although the mechanisms for dsRNA uptake from the gut lumen into the epithelia cells of insects through feeding are poorly understood [27]–[30], the silencing signal, siRNA induced by dsRNA, seems to spread from the initial entry sites via cells and tissues [27]–[30]. Thus, siRNA is transient and not synchronous in intestine tissue of insects [27]. However, cultured insect cells showed RNAi when soaked in medium with dsRNA, and the dsRNA uptake into cells was synchronous [25], [28], [31]. It may be for this reason that siRNA could be easily detected in VCMs transfected with dsRNA, but not in intact leafhoppers after ingestion of dsRNA. It is apparent that insects lack an RNA-dependent RNA polymerase (RdRp) to amplify siRNA induced by dsRNA [30]. Our results support the earlier conclusion that insects may have alternative RdRP-like mechanisms that result in systemic RNAi [25], [28]–[30].

RNAi has been extensively used to investigate the functional roles of viral proteins of reoviruses. For example, our recent findings indicated that treatment with dsRNAs against the viral gene for P9-1 of Southern rice black-streaked dwarf virus, also a plant reovirus, inhibited viral replication in its vector insect [31]. Similarly, RNAi mediated by short-interfering RNAs against viral genes has been extensively used for functional analyses of respective genome segments of animal reoviruses, which are closely related to plant reoviruses [32], [33]. Furthermore, rearrangements and reassortments of genome segments of reoviruses have long been helpful in elucidating the functions of reovirus proteins [34]–[37]. These together with RNA silencing assay have served as alternatives to plasmid-based reverse genetics.

Thus, development of RNAi induced by synthesized dsRNA, together with the system of the rearrangement or reassortment of genome segments, may help overcome the lack of a reverse genetics system for plant reoviruses and provide useful tools to investigate the molecular mechanisms enabling efficient transmission of viruses by vector insects.

Passage of persistent-propagative viruses through different organs in their vector insects requires specific interactions between virus and vector components to overcome different transmission barriers [1]–[3]. For example, the glycoproteins of tospoviruses and rhabdoviruses and the minor capisd proteins of phytoreoviruses serve as viral ligands to mediate attachment of virions to receptors on the epithelial cells of the alimentary canal of the vector insect, a necessary step for virions to overcome midgut infection barriers of vector insect [2], [11]–[13], [38]–[40]. In addition to functional viral proteins that enable the early stage interaction with host cells, persistent-propagative viruses induce the formation of various cytopathological structures involved in viral replication or cell-to-cell movement in their hosts to facilitate viral propagation. For example, tospoviruses exploit virus-containing tubules composed of a nonstructural movement protein NSm to modify plasmodesmata, allowing the transport of entire viral particles [41]. In the present study, for the first time, we have provided experimental evidence to show that a virus transmitted by its insect vector in a persistent-propagative manner can also use virus-containing tubules composed of a nonstructural protein to traffic along actin-based cellular machinery, allowing efficient cell-to-cell spread of the virus in vector insect. In this respect, the transport of virus-containing tubules along actin-based cellular machinery in vector insects resembles the extension of the tubules composed of movement proteins of viruses through plasmodesmata in host plants, suggesting that viruses evolved conserved strategies for viral intercellualr spread in vector insects or host plants.

## Materials and Methods

### Cells, viruses and antibodies

The NC-24 line of *Nephotettix cincticeps* (leafhopper) cells was maintained in monolayer culture in LBM growth medium [42]. RDV was purified from infected rice plants without the use of CCl_4_, as described by [43]. The antibodies against Pns10 and against intact viral particles (virus antigens) were described in an earlier report [14].

### Immunofluorescence staining of internal organs of leafhopper after acquisition of virus

The second-instar nymphs of *N. cincticeps* were allowed a 2-day acquisition access period (AAP) on rice plants infected with RDV. At different days after the AAP, internal organs from leafhoppers were dissected, fixed in 4% paraformaldehyde and processed for analysis of immunofluorescence as described previously [9], [14]. Pns10 tubules were immunolabeled with Pns10-rhodamine; viral particles were immunolabeled with virus-Alexa Fluor 647 or virus-FITC; actin was immunolabeled with FITC-phalloidin (Sigma). As controls, dissected organs from leafhoppers that fed on healthy plants were immunolabeled exactly in the same way. Samples were examined with a Leica TCS SP5 inverted confocal microscope essentially as described previously [9].

### dsRNA production

DNA fragment spanning a 1062-bp segment of the Pns10 gene of RDV was amplified by PCR using a forward primer (5′ *ATTCTCTAGAAGCTTAATACGACTCACTATAGGG*GAAGTAGACACTGCTACGTTTGTTCG 3′) and a reverse primer (5′ *ATTCTCTAGAAGCTTAATACGACTCACTATAGGG*GGAACCGCCGCCTTTAAG 3′), both possessing a T7 promoter (italics) at the 5′ end. The DNA fragment spanning a 717-bp segment of YFP was amplified by PCR using a forward primer (5′ *ATTCTCTAGAAGCTTAATACGACTCACTATAGGG*GTGAGCAAGGGCGAGGAGCT 3′) and a reverse primer (5′ *ATTCTCTAGAAGCTTAATACGACTCACTATAGGG*CTTGTACAGCTCGTCCATGC 3′), both possessing a T7 promoter (italics) at the 5′ end. The PCR products were used for dsRNA synthesis according to the protocol of the T7 RiboMA Express RNAi System kit (Promega). The dsRNAs were purified according to the manufacturer's instructions and checked for quality by agarose gel electrophoresis and quantified by using a spectrophotometer.

### Examination of *in vitro* spread of RDV in the presence of synthesized dsRNAs

VCMs were grown in LBM medium supplemented with 10% fetal bovine serum (FBS; Invitrogen). VCMs were transfected using lipid cellfectin using an adaptation of the manufacturer's protocol, as reported by [25]. Briefly, VCMs on a coverslip (15 mm diameter) were seeded and allowed to settle for 3 days to maintain exponential growth, and then 1 µg dsRNA was mixed with 8 µl cellfectin transfection reagent (Invitrogen) in LBM medium without FBS supplementation. The complex was incubated at room temperature for 20 min and then added to the VCMs from which normal growth medium had been removed. After a 6-h incubation, the inoculum was removed, and the coverslip was covered with LBM medium plus 10% FBS.

To gauge the effects of the dsRNAs on the direct cell-to-cell spread of RDV, after a 24-h treatment with the dsRNAs, the VCMs were inoculated with RDV at a low multiplicity of infection (MOI) of 0.001, and from 2 h p.i. onward, virus-neutralizing antibodies (30 µg/mL of medium) were added to the culture medium to inhibit infection by RDV particles that had been released into or were present in the culture medium, as described previously [14]. VCMs were fixed 3 days after viral inoculation, immunolabeled with virus-FITC and Pns10-rhodamine, and visualized by fluorescence microscopy. A minimum of four fields was examined for the foci of infection formed in three or more independent experiments.

To examine the effects of the synthesized dsRNAs on the multiplication of RDV, at 24-h after the treatment with dsRNAs, we inoculated VCMs with RDV at an MOI of 10, and cells were grown further for 72 h. After harvest, cells were subjected to several cycles of freezing and thawing to release viral particles, and lysates were stored at −70°C prior to analysis. The titer of cell-associated viruses was determined in duplicate using the fluorescent focus assay [15], [44]. Endpoint titers were calculated as means with standard deviations.

### Examination of RDV spread in the intact insect in the presence of synthesized dsRNAs

A membrane-feeding method for delivering dsRNAs to leafhoppers was used. Briefly, the second-instar nymphs of leafhopper were fed with 0.5 µg/µl dsRNAs diluted in 5% sucrose in water, which was held between two layers of stretched parafilm covering one open end of the tube. The nymphs of leafhoppers were maintained with this mixed diet for 1 day, allowed a 2-day AAP on RDV-infected rice plants, and then fed on healthy rice seedling. Total RNAs were extracted by TRIzol Reagent (Invitrogen). The effects of dsRNAs on the accumulation of the nonstructural protein Pns10 and major outer capsid protein P8 of virus were determined by RT-PCR. To determine whether blocking the formation of virus-containing Pns10 tubules would inhibit viral spread among leafhopper tissues, internal organs from leafhoppers that had received dsRNAs were fixed and processed for immunofluorescence microscopy as described earlier. To determine whether the ingestion of dsRNAs would inhibit viral transmission, we allowed the dsRNAs-treated leafhoppers a 2-day AAP on RDV-infected rice plants, then allowed them to feed on healthy rice seedling for 20 days as described previously [45]. Individual adult viruliferous insects that matured during this 20-day period were then exposed to healthy rice seedlings in individual test tubes for a 2-day inoculation access feeding. The developing leaves were scored for the first visible symptoms daily until harvest.

### Electron microscopy

For transmission electron microscopy, the internal organs from RDV-infected or healthy leafhoppers were fixed and examined as described previously [11], [14].

### siRNA detection and viral plus-strand RNAs detection by Northern blot analysis

The basic procedure to detect siRNA was that of [46]. Briefly, at 72 h after the treatment with dsRNAs, VCMs were harvested, and total RNA was extracted with TRIzol Reagent (Invitrogen). DIG-labeled negative-sense RNA transcripts of Pns10 or YFP genes were generated *in vitro* with T7 RNA polymerase using a DIG RNA Labeling kit (Roche), then used as a probe. Northern blots were produced using a DIG Northern starter kit (Roche) and standard protocols.

Viral plus-strand RNAs were analyzed by northern blot hybridization using a DIG Northern starter kit (Roche) according to standard protocols. VCMs transfected with dsRNAs were inoculated with RDV at an MOI of 10, and cells were further cultured for 72 h. Second-instar nymphs of leafhopper were fed with dsRNAs for 1 day, allowed a 2-day AAP on RDV-infected rice plants, then fed on healthy rice seedling for 10 days. Total RNAs from VCMs or intact leafhoppers were extracted with TRIzol Reagent (Invitrogen). DIG-labeled negative-sense RNA transcripts of Pns10 or P8 genes were generated *in vitro* with T7 RNA polymerase using a DIG RNA Labeling kit (Roche), then used as a probe. Northern blots were produced using a DIG Northern starter kit (Roche) and standard protocols.

### RT-qPCR assay

The effects of RNAi induced by dsPns10 on the synthesis of viral plus-strand RNAs were further analyzed by RT-qPCR assay, essentially as described previously [47], [48]. Briefly, VCMs transfected with dsRNAs were inoculated with RDV at an MOI of 10, and cells were further cultured for 72 h. Total RNAs from VCMs were extracted with TRIzol Reagent (Invitrogen). RT-qPCR primers from the sequences of P8 and Pns10 genes of RDV were designed and tested for efficiency and specificity. RT-qPCR assay was carried out in a Mastercycler realplex4 real-time PCR system (Eppendorf) using the SYBR Green PCR Master Mix kit (QIAGEN) according to standard protocol. The level of leafhopper actin gene was used as the internal control for each RT-qPCR assay. Relative to actin gene was used for quantitative analysis using the Microsoft Excel based tools.

## Supporting Information

Figure S1 **Transmission electron micrographs showing the association of virus-containing tubules with microvilli of the alimentary canal in viruliferous leafhoppers.** The presence of virus-containing tubules (arrows) in microvilli and lumen of filter chamber (**A**), anterior midgut (**B**) and middle midgut (**C**). Images I and II in panel **C** show tubules of different lengths in the microvilli of middle midgut. Image III in panel **C** is a transverse section of tubule-associated microvillus of middle midgut. EC, epithelial cell. GL, gut lumen. Mv, microvilli. Bars, 200 nm.
(TIFF)
