# Supplementary figures and images for: Tubular Structure Induced by a Plant Virus Facilitates Viral Spread in Its Vector Insect
Source: PLoS Pathog. 2012 Nov 15;8(11):e1003032. doi: 10.1371/journal.ppat.1003032 (PMC3499585; doi:10.1371/journal.ppat.1003032)

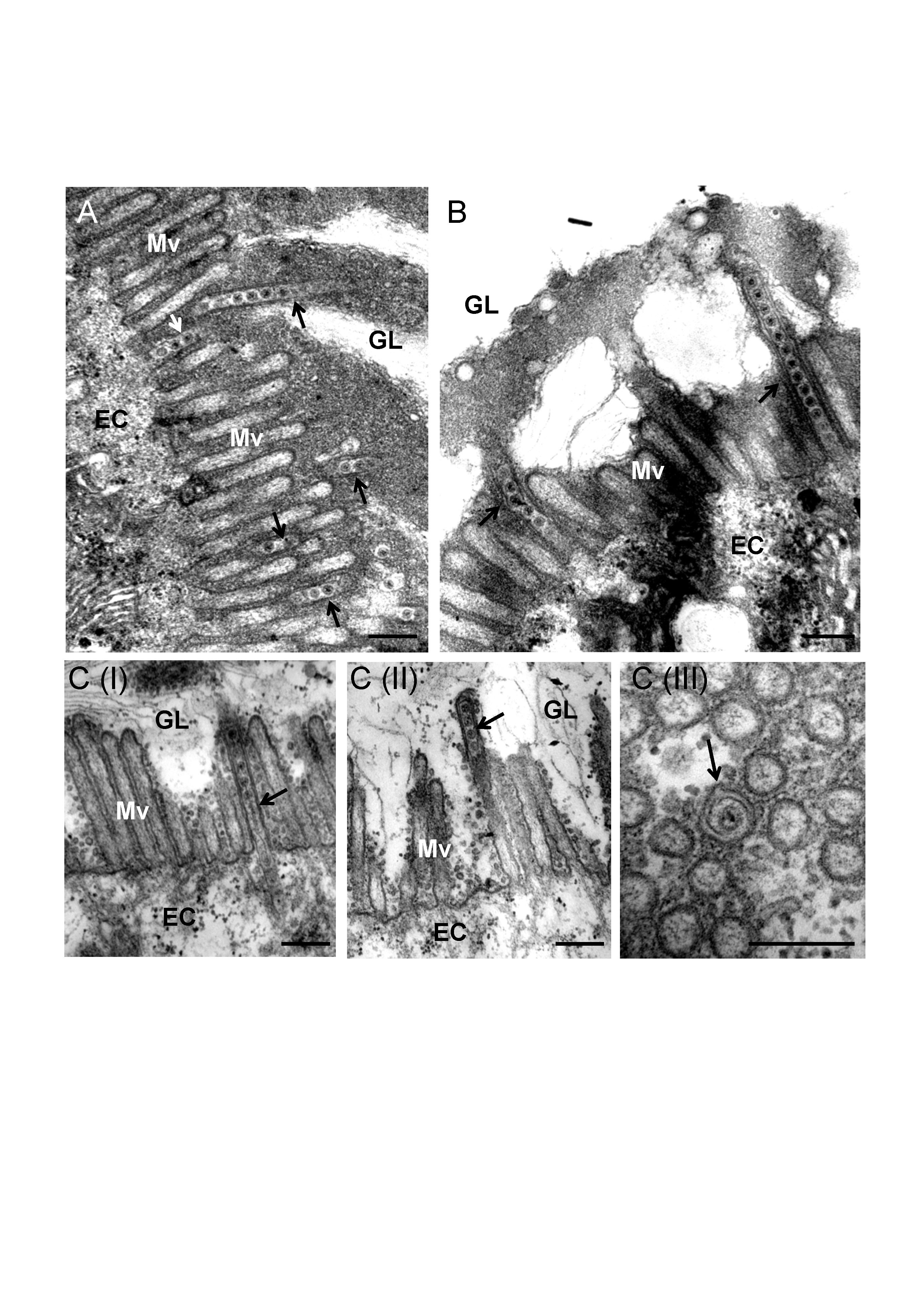

Supplement: Figure S1 — Transmission electron micrographs showing the association of virus-containing tubules with microvilli of the alimentary canal in viruliferous leafhoppers. The presence of virus-containing tubules (arrows) in microvilli and lumen of filter chamber (A), anterior midgut (B) and middle midgut (C). Images I and II in panel C show tubules of different lengths in the microvilli of middle midgut. Image III in panel C is a transverse section of tubule-associated microvillus of middle midgut. EC, epithelial cell. GL, gut lumen. Mv, microvilli. Bars, 200 nm. (TIFF) [file ppat.1003032.s001.tif]
